# PARP1 inhibitor (PJ34) improves the function of aging-induced endothelial progenitor cells by preserving intracellular NAD^+^ levels and increasing SIRT1 activity

**DOI:** 10.1186/s13287-018-0961-7

**Published:** 2018-08-23

**Authors:** Siyuan Zha, Zhen Li, Qing Cao, Fei Wang, Fang Liu

**Affiliations:** Department of Geriatrics, Xinhua Hospital, School of Medicine, Shanghai Jiao Tong University, Shanghai, China

**Keywords:** Endothelial progenitor cells, Senescence, Poly (ADP-ribose) polymerase 1, Nicotinamide adenine dinucleotide, Sirtuin 1

## Abstract

**Background:**

Nicotinamide adenine dinucleotide (NAD^+^) is a critical molecule involved in various biological functions. Poly (ADP-ribose) polymerase 1 (PARP1) and sirtuin 1 (SIRT1) affect cellular NAD^+^ levels and play essential roles in regulating metabolism. However, there has been little research on the effects of PARP1 and SIRT1 crosstalk during senescence.

**Methods:**

We isolated endothelial progenitor cells (EPCs) from human umbilical cord blood and treated them with a PARP1 inhibitor (PJ34).

**Results:**

Using a stress-induced premature aging model built by H_2_O_2_, transfection with adenoviral vectors, and Western blot analysis, we observed that PJ34 treatment preserved intracellular NAD^+^ levels, increased SIRT1 activity, decreased p53 acetylation, and improved the function of stress-induced premature aging EPCs.

**Conclusions:**

Our results suggest that PJ34 improves the function of aging-induced EPCs and may contribute to cellular therapies for atherosclerosis.

**Electronic supplementary material:**

The online version of this article (10.1186/s13287-018-0961-7) contains supplementary material, which is available to authorized users.

## Background

Atherosclerosis (AS) is a major cause of cardiovascular disease, and endothelial injury and dysfunction caused by aging are classical risk factors for AS [1–3]. In recent years, several studies have revealed that endothelial progenitor cells (EPCs) play a crucial role in the replacement of injured vascular endothelial cells. Patients with AS display a significant decrease in the number of EPCs in their peripheral blood [4]. Animal experiments have demonstrated that EPCs can repair injured endothelial cells and improve angiogenesis during ischemia [5]. Further research showed that the ability of EPCs to replace impaired endothelial cells depends on their number and functionality [6]. However, EPC function gradually degenerated with senescence, showing decreased cellular viability, slower migration, and degressive angiogenic ability [7–10]. Therefore, improving the function of aging EPCs may aid in the prevention of atherosclerosis and other vascular diseases caused by endothelial damage.

Nicotinamide adenine dinucleotide (NAD^+^) is a critical molecule involved in various biological functions such as energy generation, metabolism, and DNA repair. NAD^+^ also plays an important role during aging because it participates in oxidation-reduction (redox) reactions in the tricarboxylic acid (TCA) cycle [11–14].

Poly (ADP-ribose) polymerase 1 (PARP1) is a genome-stabilizing enzyme that catalyzes the covalent transfer of mono- or poly-adenosine diphosphate (ADP) units from NAD^+^ to glutamate or aspartate residues within target proteins, resulting in protein-conjugated chains of poly ADP-ribose (PAR) polymers [15]. PARP1 is involved in DNA repair and, in the presence of pathological DNA damage, its activity can result in excessive NAD^+^ consumption [16–18].

Sirtuin 1 (SIRT1) is a redox-sensitive protein involved in a wide range of cellular processes, including aging, oxidative stress responses, metabolism, circadian rhythm regulation, and proliferation [19–22]. It is a NAD^+^-dependent deacetylase that targets several transcription factors, including forkhead box O3, tumor protein p53, nuclear factor κB, and peroxisome proliferator-activated receptor γ coactivator 1 alpha. Therefore, its activity plays an important role in health maintenance [23, 24].

PARP1 and SIRT1 affect NAD^+^ levels and play crucial roles in regulating cellular metabolism [25]. However, there is little research on the effects of PARP1 and SIRT1 crosstalk on senescence. To study its effects on aging EPCs, we treated EPCs obtained from human umbilical cord blood with a PARP1 inhibitor (PJ34) and assayed the effects of PARP1 and SIRT1 activity on EPC senescence.

PJ34, a PARP inhibitor, can inhibit the activation from PARP to PAR and thereby preserve intracellular NAD^+^ levels, which improves mitochondrial function [26].

## Methods

### Study design

The overall objective of this study was to determine whether PJ34 is able to inhibit EPC senescence and, if so, the mechanism. Firstly, we used H_2_O_2_ to build a stress-induced premature aging model [27]. Endogenous DNA damage induced by sublethal oxidative stress is responsible for the initiation and progression of the senescent phenotype [28]. According to the changes of H_2_O_2_-activated PAR and the cellular states observed under a microscope, we selected an optimum concentration of H_2_O_2_. Furthermore, we used several concentrations of PJ34 to inhibit the activation of PARP and selected an optimum concentration on the basis of changes in PAR. Next, we established four treatments to study the effects of PJ34 on aging EPCs. Cells treated with PJ34 were used to measure the effects of PJ34 on young EPCs, cells treated with H_2_O_2_ were used to induce premature cellular senescence, and cells treated with H_2_O_2_ + PJ34 were compared with the cells treated with H_2_O_2_ to verify the effects of PJ34 on aging EPCs. Untreated cells were used as a negative control. Finally, we used SIRT1 short-hairpin RNA (Ad-sh-SIRT1) to silence SIRT1 expression in EPCs and measured the effects of PJ34 again. We measured and evaluated PARP1 activity by assaying the production of PAR [15, 26]. SIRT1 activity was evaluated by analyzing p53 acetylation [29, 30]. EPC functionality changes with senescence were evaluated by a series of functional experiments. For example, senescence-associated beta-galactosidase (SA-β-gal) staining was used to identify cellular senescence directly [31, 32], cell counting kit (CCK)-8 was used to analyze cell viability [7], transwell trials were used to observe cell migration [8, 9], and Matrigel angiogenesis assays were used to determine the angiogenic ability [10].

### EPC isolation and culture

The Ethics Committee of Xinhua Hospital Affiliated to Shanghai Jiao Tong University School of Medicine approved this study. EPCs were isolated from human umbilical cord blood by density gradient centrifugation with Histopaque-1077 (Sigma). We gently added human blood to the separation solution and centrifuged at 2000 rpm for 20 min at 4 °C. After density gradient centrifugation, the uppermost layer contains the serum, while the lowermost layer is composed of red blood cells, and the middle layer of white blood cells is suspended in the separation liquid. We withdrew the white blood cells, resuspended these in complete endothelial cell growth medium (EGM)-2 (Lonza), and then seeded them in six-well plates. Cells from approximately 10 mL of cord blood were plated per well. The medium was changed every 3 days, and cells were subcultured at a 1:3 ratio. EPC endothelial markers were detected by immunofluorescence and flow cytometry. For immunofluorescence, antibodies against CD31, CD34, von Willebrand factor (vWF), vascular endothelial growth factor receptor 2 (VEGFR2), and CD133 were purchased from Cell Signaling Technology. For flow cytometry, antibodies against CD34, CD133, and VEGFR2 were purchased from Invitrogen.

### Western blot analysis

Cellular proteins were extracted using radioimmunoprecipitation assay buffer, and protein concentrations were measured using the bicinchoninic acid method. Approximately 30 μg of protein per well was loaded on 8 or 10% gels for sodium dodecyl sulfate-polyacrylamide gel electrophoresis. Proteins were transferred to polyvinylidene difluoride (PVDF) membranes (Millipore), and sequentially detected by primary antibodies, secondary antibodies, and enhanced chemiluminescence (Millipore). Antibodies against PARP1, SIRT1, acetylated (ac)-p53, p53, and cyclin-dependent kinase inhibitor 1A (p21), as well as anti-mouse and anti-rabbit secondary antibodies, were purchased from Cell Signaling Technology. The anti-PAR antibody was purchased from Invitrogen. An anti-β-actin antibody (Cell Signaling Technology) was used as an internal control.

### Cell viability assays

CCK-8 (Dojindo) was used to analyze cell viability after various treatments. Cells (1 × 10^4^/well) were suspended in 100 μL fresh medium containing the various treatments and then seeded. After treatment, CCK-8 (10 μL/well) was added for 3 h of additional incubation, and the absorbance was measured at 450 nm.

### Migration assays

Transwell plates (Corning) were used to observe cell migration. Complete fresh EGM-2 containing the various treatments (600 μL) was added to the bottom chambers, and 5 × 10^4^ cells suspended in 200 μL serum-free medium containing the various treatments were added to the top chamber. After incubation at 37 °C for 12 h, transmigrated cells were fixed in 4% paraformaldehyde and stained with crystal violet. Three random microscopic fields were selected, and the stained cells were counted.

### Matrigel angiogenesis assays

Matrigel™ (50 μL; BD Biosciences) was added to the wells of 96-well plates and incubated at 37 °C for 30 min. Then, 2 × 10^4^ cells/well were seeded on the Matrigel and incubated at 37 °C. Images were acquired after 8 h.

### SA-β-Gal assays

An SA-β-gal staining kit (Cell Signaling Technology) was used to identify cellular senescence. Cells were fixed for 20 min at room temperature and then incubated in staining solution overnight at 37 °C. Three random microscopic fields were selected, and the stained cells were counted.

### Cellular reactive oxygen species (ROS) measurements

The reactive oxygen species assay kit (Sigma) was used to measure cellular ROS. Cells were seeded in a 96-well plate and incubated with 10 μM dichloro-dihydro-fluorescein diacetate (DCFH-DA) at 37 °C for 30 min. Cells were washed with phosphate-buffered saline three times, and then incubated in fresh medium containing the various treatments. After treatment, the absorbance was measured using a fluorescence enzyme-labeling device at excitation and emission wavelengths of 485 and 535 nm, respectively.

### NAD^+^ measurements

The NAD/NADH assay kit (Abcam) was used to measure NAD/NADH levels. The standard solution was prepared according to the manufacturer’s protocol, and the NAD/NADH concentration was calculated using the standard curve. The NAD^+^ concentration was calculated according to the formula NAD^+^ = total NADH − NAD.

### Adenovirus transfection

Adenoviral vectors containing green fluorescent protein (Ad-GFP) and Ad-sh-SIRT1 were purchased from Hanheng Biotechnology. Ad-GFP was used as a control. Cells were transfected for 6 h and then incubated with fresh medium for 48 h, after which protein expression levels were analyzed by Western blot, or additional treatments were performed.

### Statistical analysis

The results are expressed as the mean ± standard error. Comparisons between two groups were performed using the independent samples *t* test. *P* values < 0.05 were considered statistically significant. All experiments were performed independently in triplicate at a minimum.

## Results

### Identification of EPCs and analysis of their protein expression

**Fig. 1:**
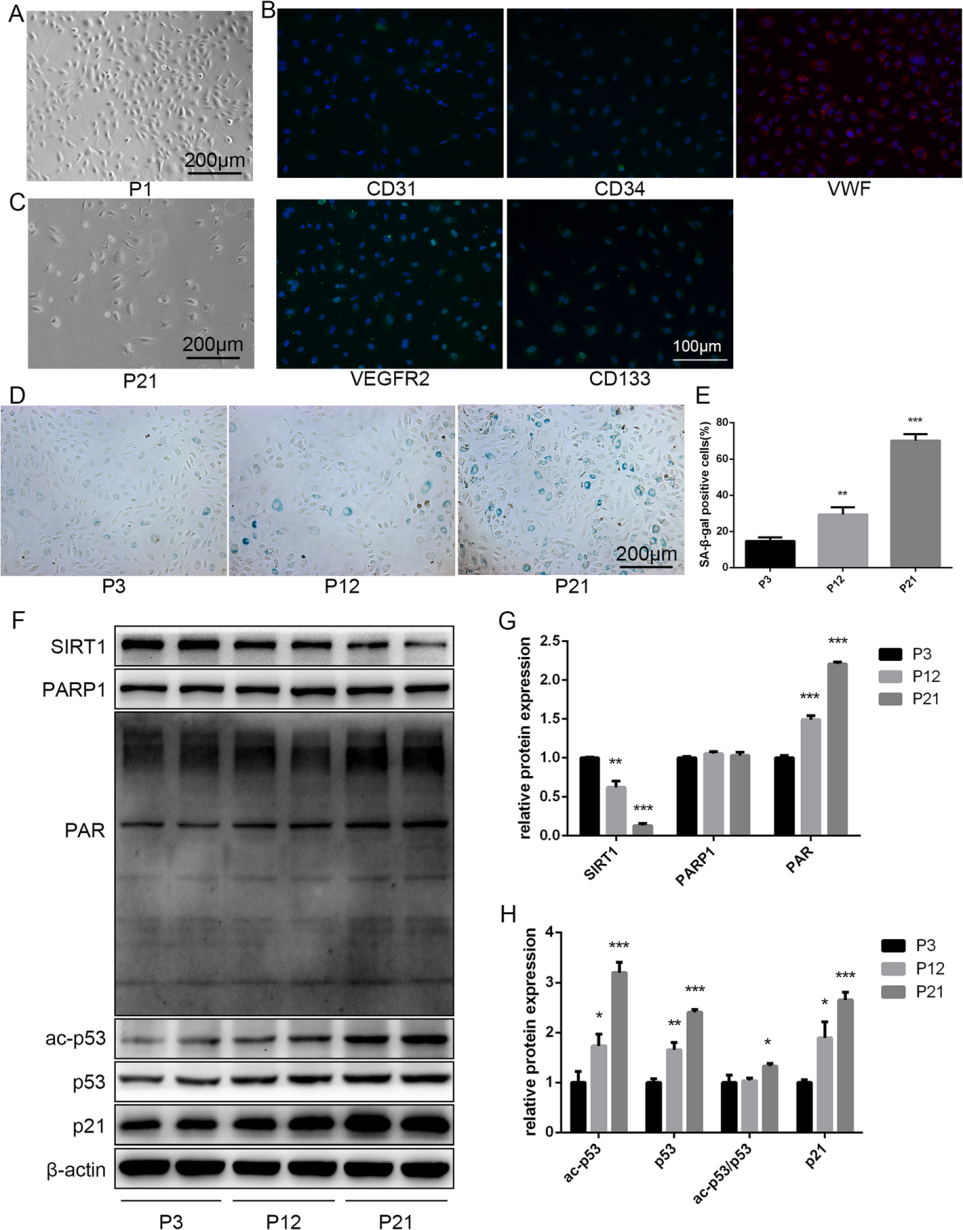
Identification of EPCs from human umbilical cord blood and protein expression during replicative aging. **a** EPCs (passage (P)1) after 2 weeks (100×). **b** Cells were characterized by immunofluorescence detection of CD31, CD34, von Willebrand factor (VWF), vascular endothelial growth factor receptor 2 (VEGFR2), and CD133. **c** Morphology of aging EPCs (100×). **d**, **e** Senescence-associated beta-galactosidase (SA-β-gal) staining at P3, P12, and P21. **f**–**h** Protein levels of poly (ADP-ribose) polymerase 1 (PARP1), poly ADP-ribose (PAR), sirtuin 1 (SIRT1), acetylated (ac)-p53, p53, and p21 during replicative senescence. *P < 0.05, **P < 0.01, ***P < 0.001, versus the control

The isolated cells exhibited monolayer growth and cobblestone morphology, like typical EPCs (Fig. 1a). Most of the cells expressed CD31, CD34, vWF, VEGFR2, and CD133, which are considered typical markers of EPCs (Fig. 1b) [33]. Flow cytometry analysis revealed that the positive expression rates of CD34, VEGFR2, and CD133 were 90.8%, 93.8%, and 95.0%, respectively (Additional file 1: Figure S1). VEGFR2 was considered as a classical endothelial cell marker. CD34 and CD133 were considered as classical progenitor cell markers [34].

To investigate the differences between young and senescent EPCs, we repeatedly subcultured EPCs for up to 21 passages (to P21). Senescence was confirmed through morphology and SA-β-gal assays. Cells at P1 were small and displayed typical cobblestone-like morphology. With repeated subculture, P21 cells became larger and more irregular in shape, and some were branch-like and polygonal or long and spindle-shaped (Fig. 1c). SA-β-gal positivity increased with repeated subculture (Fig. 1d, e).

We next examined the levels of SIRT1, PARP1, PAR, ac-p53, p53, and p21. Our results showed that with the repeated subculture SIRT1 decreased, while PAR, ac-p53, p53, and p21 increased. There was no significant difference in PARP1 levels between young and replicative aging EPCs (Fig. 1f–h).

### Effects of H_2_O_2_ and PJ34 on protein expression

**Fig. 2:**
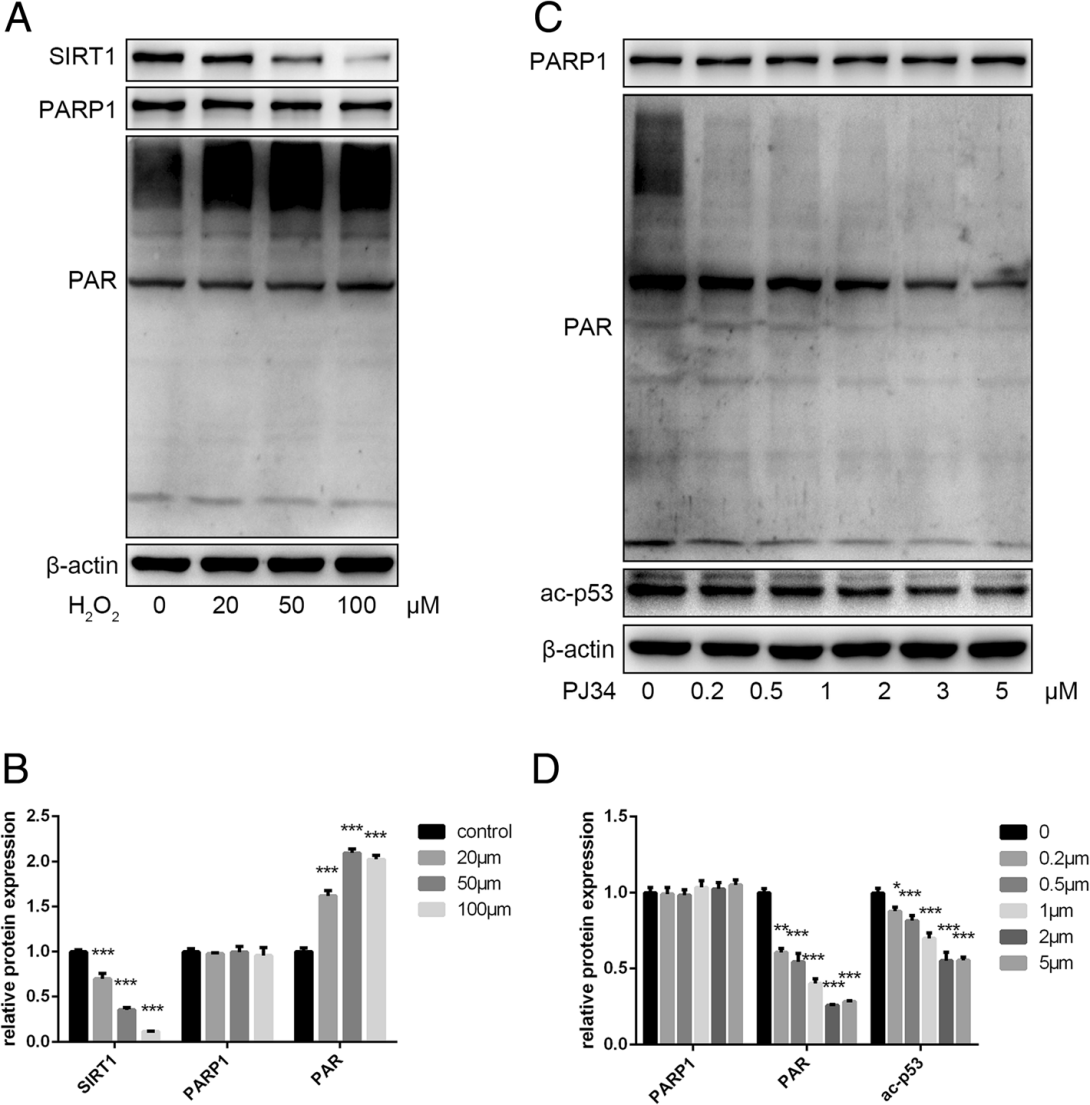
Effects of H_2_O_2_ and PJ34 treatment on EPC protein expression. **a**, **b** Expression of sirtuin 1 (SIRT1), poly (ADP-ribose) polymerase 1 (PARP1), and poly ADP-ribose (PAR) as analyzed by Western blot. **c**, **d** Expression of PARP1, PAR, and acetylated (ac)-p53 as analyzed by Western blot. *P < 0.05, **P < 0.01, ***P < 0.001, versus the control

To verify the effects of crosstalk between PARP1 and SIRT1 on senescence, we first established a stress-induced premature aging model by treating young EPCs (P3–P5) with H_2_O_2_, and then used various concentrations of PJ34 to inhibit PARP1 activation. By Western blot, we found that H_2_O_2_ decreased SIRT1 levels and increased PAR synthesis by activating PARP1 (Fig. 2a, b). PJ34 inhibited PARP1 activation, resulting in decreased PAR synthesis, which may preserve intracellular NAD^+^ levels during cellular senescence (Fig. 2c, d).

### Effects of PJ34 on aging EPCs

To further verify the effects of crosstalk between PARP1 and SIRT1 on senescence, we first determined the proper concentrations of H_2_O_2_ and PJ34. EPCs (P3–P5) were incubated with normal fresh culture medium, 2 μM PJ34, 100 μM H_2_O_2_, or 2 μM PJ34 + 100 μM H_2_O_2_ for 6 h. After incubation, the culture medium was removed. Cells treated with PJ34 were incubated in fresh culture medium containing 2 μM PJ34 for 18 h, and cells subjected to other treatments were incubated in normal fresh culture medium for 18 h.

**Fig. 3:**
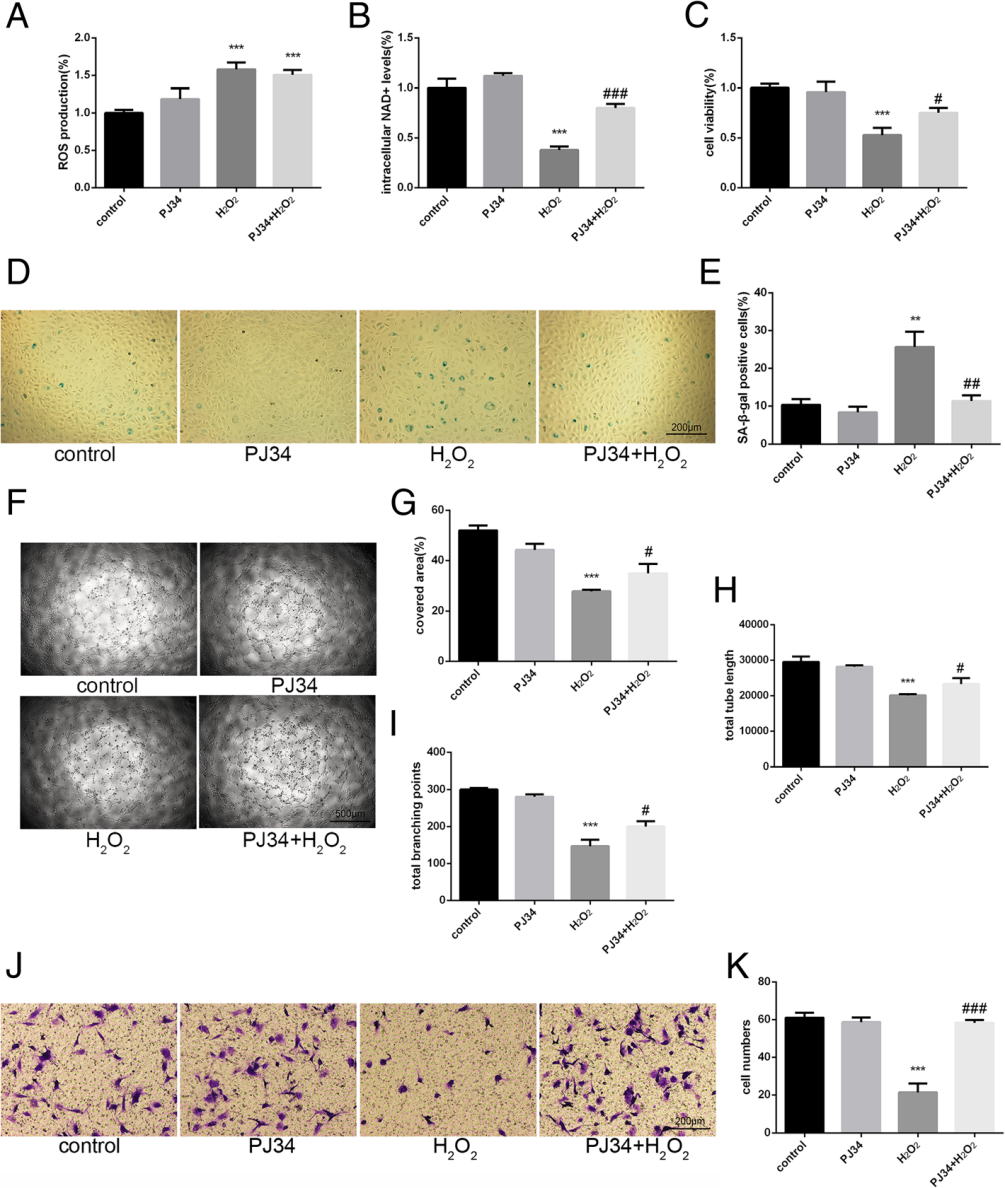
Functional alterations in senescent EPCs after PJ34 treatment. **a** Reactive oxygen species (ROS) production was assessed by DCFH-DA staining after 6 h of treatment. **b** Intracellular nicotinamide adenine dinucleotide (NAD^+^) levels were measured after 6 h of treatment. **c** Cell viability was evaluated by CCK-8 after 24 h of treatment. **d**, **e** Senescence was analyzed by senescence-associated beta-galactosidase (SA-β-gal) staining after 24 h of treatment. **f**–**i** Tube formation ability was evaluated on Matrigel after 24 h of treatment. **j**, **k** Migration ability was analyzed by Transwell assay after 24 h of treatment. *P < 0.05, **P < 0.01, ***P < 0.001, versus the control; ^#^*P* < 0.05, ^##^*P* < 0.01, ^###^*P* < 0.001, versus H_2_O_2_ treatment

ROS production increased 6 h after H_2_O_2_ treatment (Fig. 3a). In addition, intracellular NAD^+^ levels decreased markedly in cells treated with H_2_O_2_ alone. However, the intracellular NAD^+^ levels of cells treated with H_2_O_2_ + PJ34 were relatively maintained, although they did decrease compared with the control (Fig. 3b). To investigate the effects of increased SIRT1 activity on aging EPCs, we measured several indicators related to senescence, including cell viability, SA-β-gal activity, tube formation ability, and migration ability. The cell viability, tube formation ability, and migration ability of cells treated with H_2_O_2_ decreased significantly but were partially restored in cells treated with H_2_O_2_ + PJ34. The rate of SA-β-gal positivity markedly increased in cells treated with H_2_O_2_, and this increase was attenuated in cells treated with H_2_O_2_ + PJ34 (Fig. 3c–k).

**Fig. 4:**
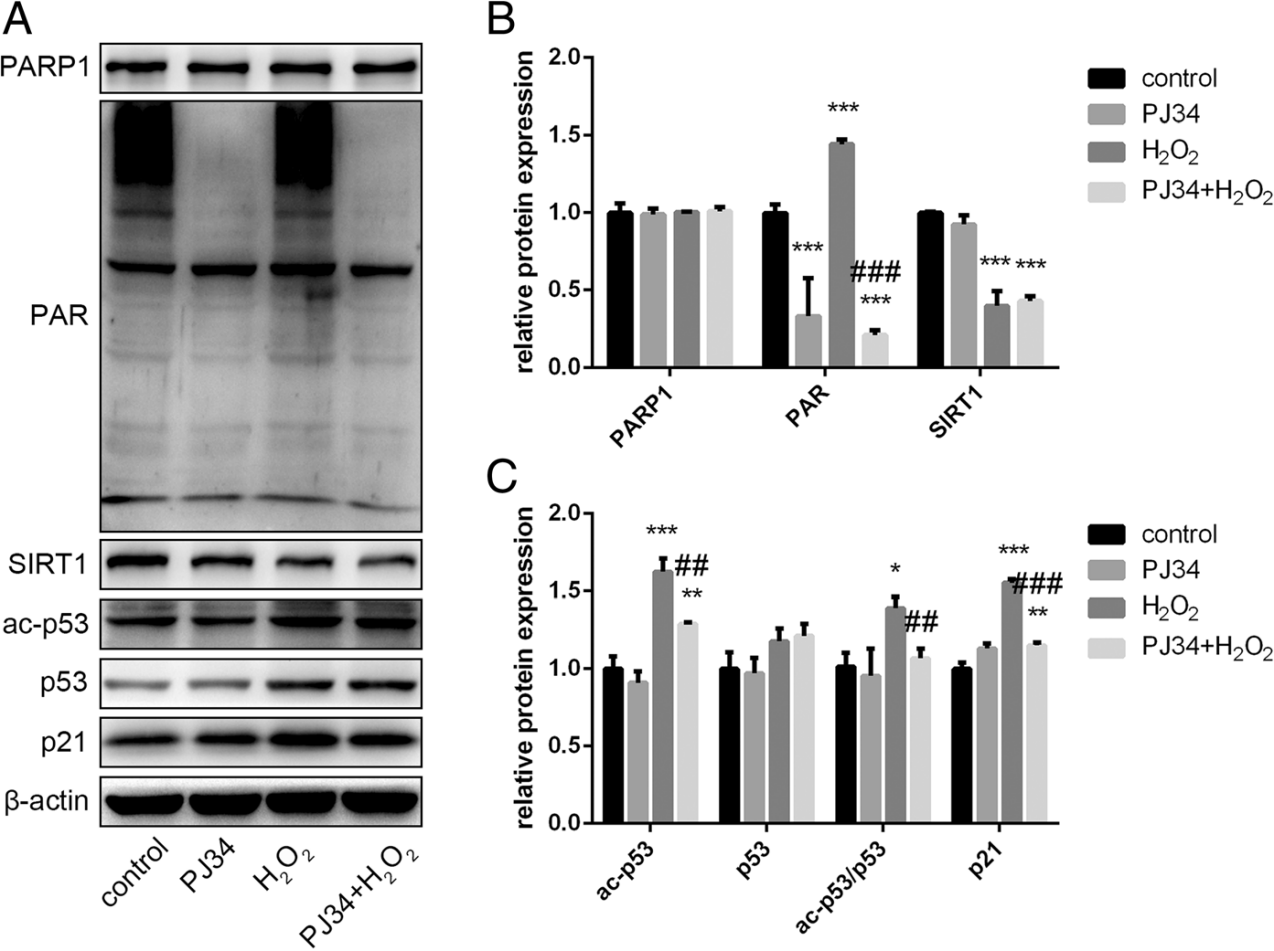
Protein expression changes in senescent EPCs with PJ34 treatment. **a**–**c** Poly (ADP-ribose) polymerase 1 (PARP1), poly ADP-ribose (PAR), sirtuin 1 (SIRT1), acetylated (ac)-p53, p53, and p21 were detected by Western blot after 24 h of treatment. *P < 0.05, **P < 0.01, ***P < 0.001, versus the control; ^#^*P* < 0.05, ^##^*P* < 0.01, ^###^*P* < 0.001, versus H_2_O_2_ treatment

To further examine the effects of PJ34, we used Western blot analysis to confirm the alteration in SIRT1 activity. In the two groups of cells treated with PJ34, PARP1 levels were barely changed but PAR levels decreased markedly. SIRT1 expression levels were no different between the two groups of cells treated with H_2_O_2_, but ac-p53 expression levels and the ac-p53/p53 ratio declined significantly after PJ34 treatment. Increased p21 levels were consistent with increased ac-p53 activity (Fig. 4). Therefore, we suggest that PJ34 activates SIRT1 by inhibiting PARP1 activation and preserving intracellular NAD^+^ levels in EPCs, and possibly reverses the effects of aging.

### Mechanism of PJ34 action in aging EPCs

**Fig. 5:**
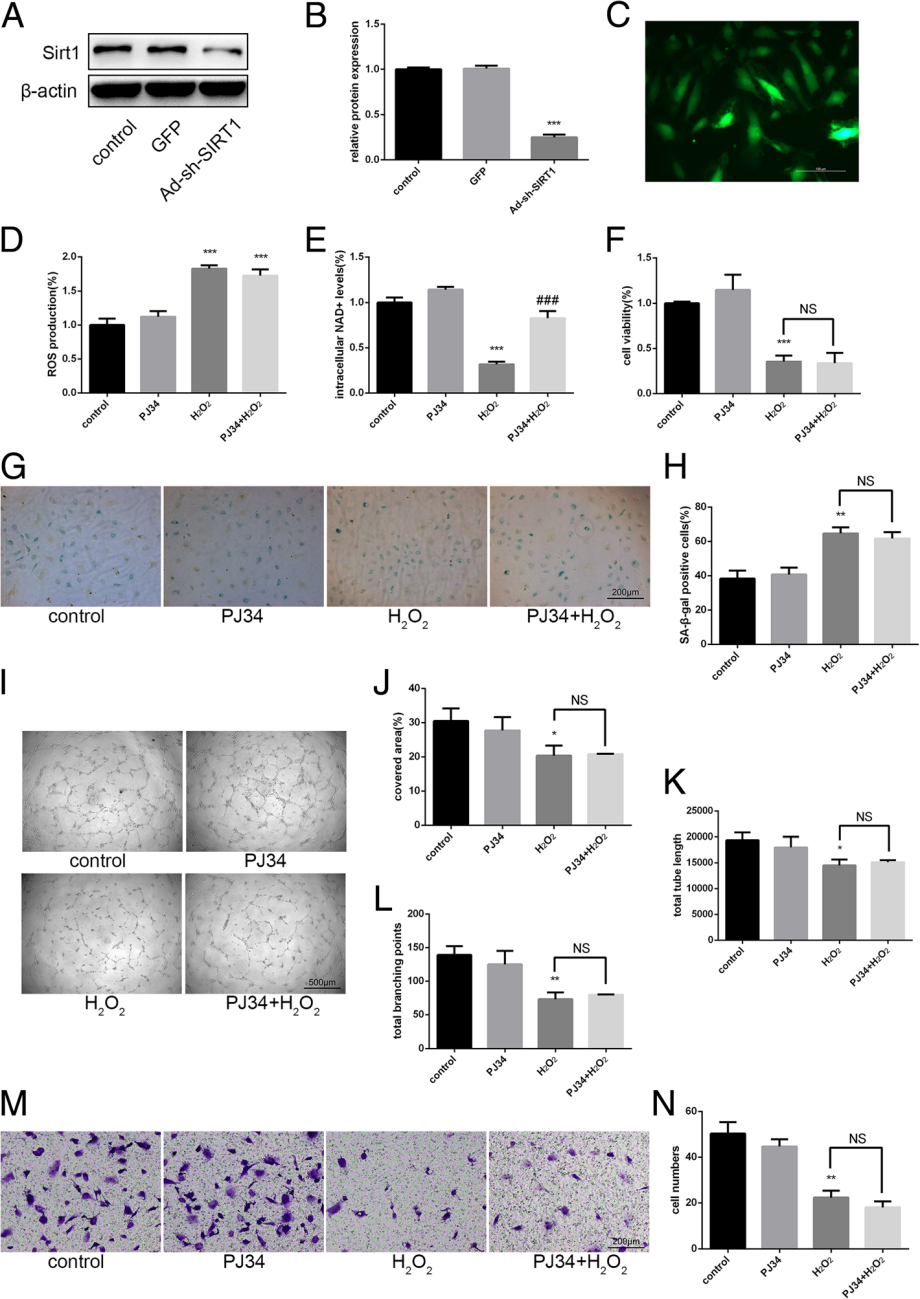
Effects of PJ34 on senescent EPC functionality after silencing sirtuin 1 (SIRT1). **a**–**c** SIRT1 expression was detected by Western blot after 48 h of SIRT1 short-hairpin RNA (Ad-sh-SIRT1) transfection. **d** Reactive oxygen species (ROS) production was assessed by DCFH-DA staining. **e** Intracellular nicotinamide adenine dinucleotide (NAD^+^) levels were measured. **f** Cell viability was evaluated via the CCK-8 assay. **g**, **h** Senescence was analyzed by senescence-associated beta-galactosidase (SA-β-gal) staining. **i**–**l** Tube formation ability was evaluated by Matrigel assay. **m**, **n** Migration ability was analyzed by Transwell assay. *P < 0.05, **P < 0.01, ***P < 0.001, versus the control; ^#^*P* < 0.05, ^##^*P* < 0.01, ^###^*P* < 0.001, versus H_2_O_2_ treatment. NS not significant

**Fig. 6:**
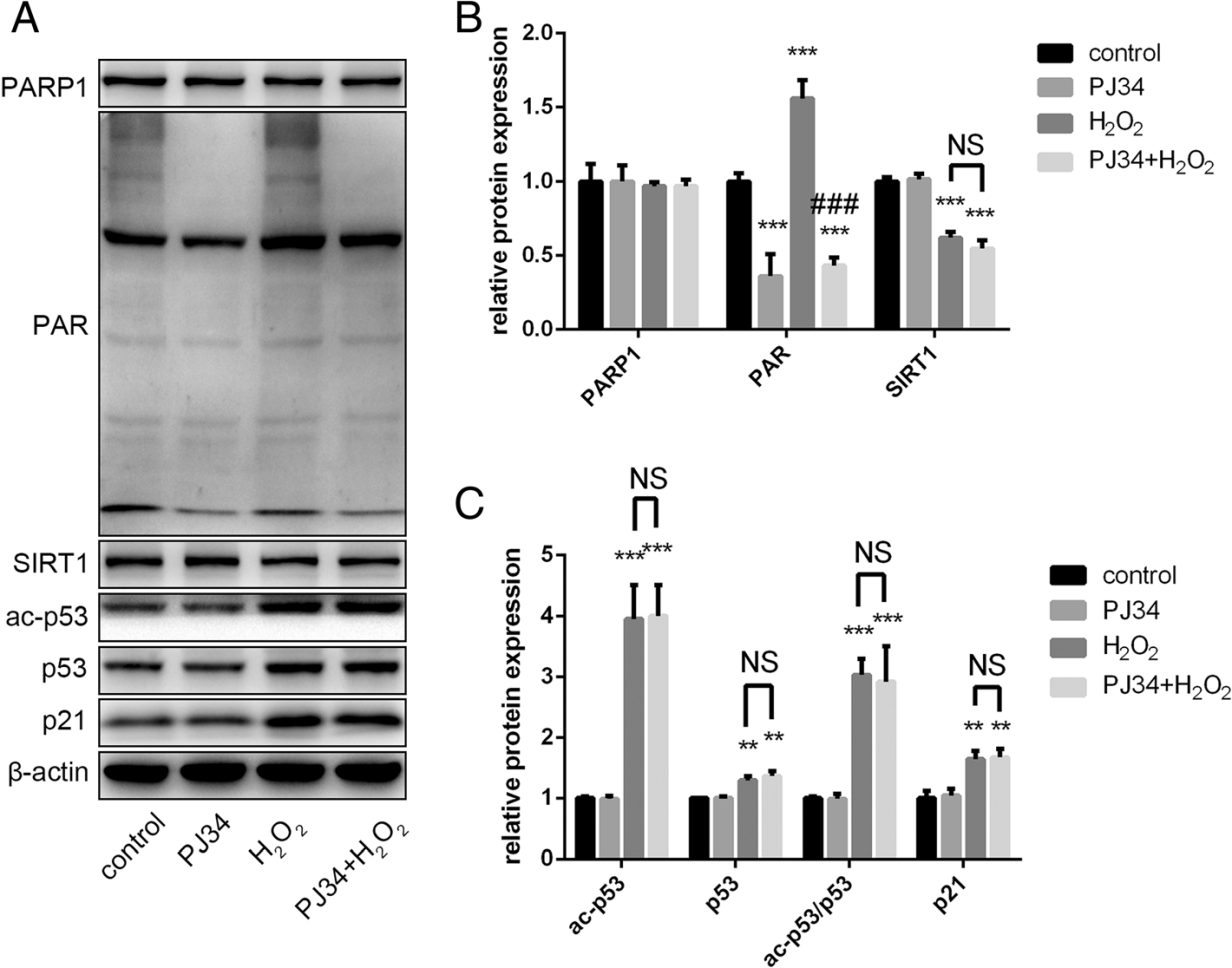
Effects of PJ34 on the protein levels of senescent EPCs after silencing sirtuin 1 (SIRT1). **a**–**c** Poly (ADP-ribose) polymerase 1 (PARP1), poly ADP-ribose (PAR), SIRT1, acetylated (ac)-p53, p53, and p21 were detected by Western blot. *P < 0.05, **P < 0.01, ***P < 0.001, versus the control; ^#^*P* < 0.05, ^##^*P* < 0.01, ^###^*P* < 0.001, versus H_2_O_2_ treatment. NS not significant

To further verify the mechanism of PJ34 in aging EPCs, we used Ad-sh-SIRT1 to attenuate SIRT1 expression (Fig. 5a–c). After 48 h, we treated cells with H_2_O_2_ and PJ34 as above. ROS production change and the effects of PJ34 on the preservation of intracellular NAD^+^ levels were again observed (Fig. 5d, e). However, the effects of PJ34 on reversing aging EPC functionality almost completely disappeared (Fig. 5f–n). By Western blot, we found that the effects of PJ34 on PARP1 inhibition remained but the previously observed decreases in ac-p53 and p21 levels were not evident (Fig. 6). Therefore, PJ34 may improve the function of aging EPCs through PARP1 inhibition, preservation of intracellular NAD^+^ levels, and increased SIRT1 activity without increased SIRT1 expression.

## Discussion

Our results suggested that SIRT1 levels decreased and P53 levels increased with replicative senescence, which is consistent with the results of previous studies [35, 36]. H_2_O_2_ promoted PARP1 to PAR activation. At the same time, H_2_O_2_ decreased SIRT1 expression and increased P53 expression; these results were similar to those of previous studies [28, 30]. Moreover, our results suggested that a certain concentration of PJ34 may revert the decreased functionality of aging-induced EPCs through SIRT1. In this process, PJ34 inhibits the PARP1 activation and thereby preserves intracellular NAD^+^ levels. Interestingly, SIRT1 expression levels were not changed, but its activity was enhanced. Therefore, with respect to the NAD^+^-dependent deacetylase (SIRT1), we speculated that increased NAD^+^ levels, as a cofactor of SIRT1, were unable to promote SIRT1 expression but activated the biological activity of SIRT1, which could be observed by the improvement of deacetylation functionality.

**Fig. 7:**
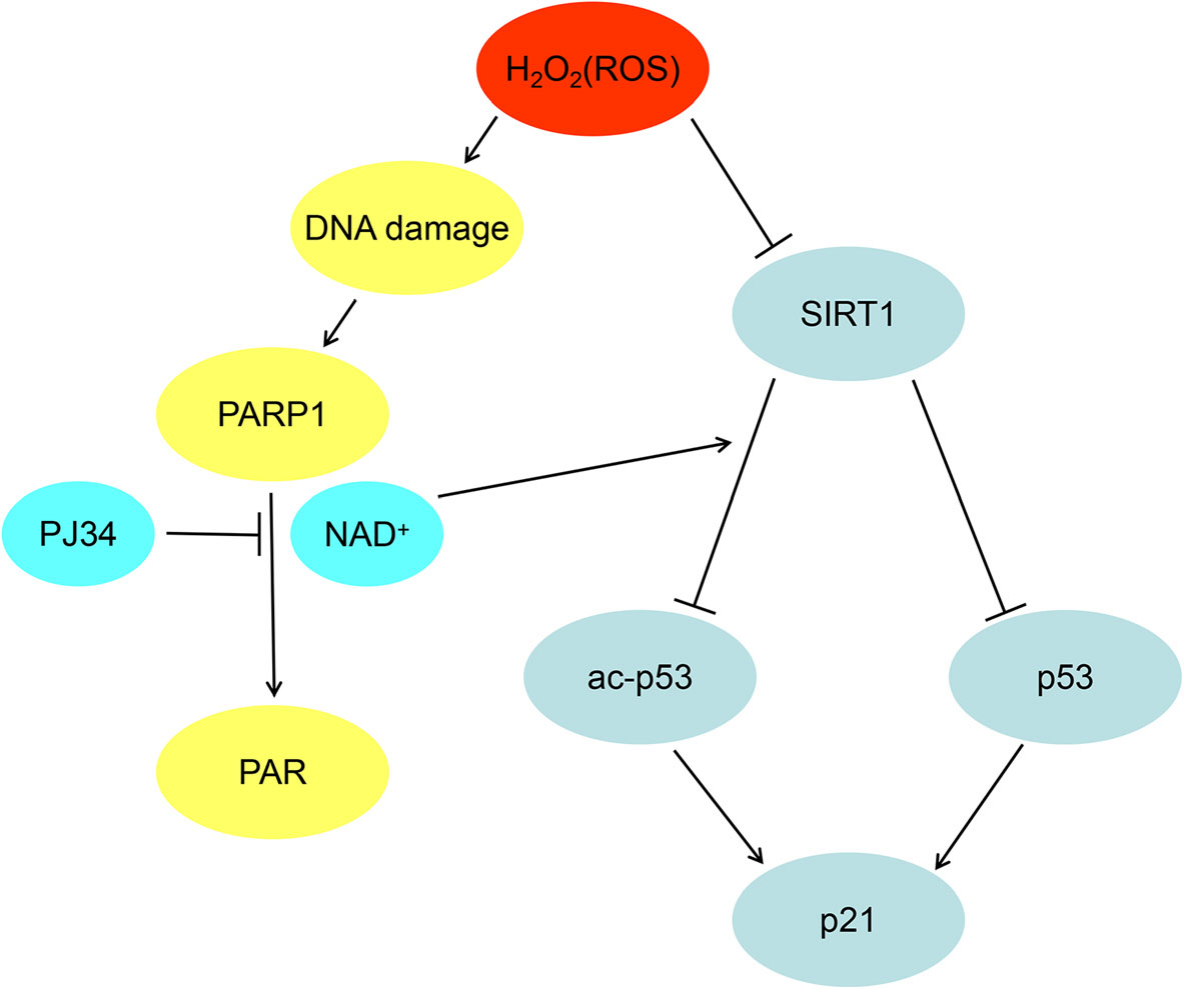
Schematic illustration of poly (ADP-ribose) polymerase 1 (PARP1) and sirtuin 1 (SIRT1) crosstalk. In EPCs, H_2_O_2_ results in DNA damage, PARP1 activation, and consequent nicotinamide adenine dinucleotide (NAD^+^) consumption. PJ34 preserves intracellular NAD^+^ levels and increases SIRT1 deacetylation. ROS reactive oxygen species

There are some advantages to our study. First, we used a novel approach to understand the effects and mechanism by treating EPCs with PJ34, which will aid further research on senescent EPCs (Fig. 7). Improvement of the functionality of aging EPCs may contribute to the development of cellular therapies for AS. Furthermore, we found that EPC functionality changed with senescence and treatment in various aspects. PAR, whose molecular weight was from 2 kD to 300 kD as a protein polymer, was very special. We demonstrated changes in its expression by Western blot.

There are several limitations to our study. Firstly, EPCs cultured from human umbilical cord blood entered a senescent stage after a limited number of cell divisions, which was identified as replicative senescence in our study [37, 38]. In previous experiments, we found that EPCs entered this stage at approximately P20. EPCs incubated with 100 μM H_2_O_2_ for 6 h and then with fresh culture medium for 18 h exhibited the same state as P20 EPCs, which were identified as being in stress-induced premature cellular senescence [32]. Therefore, we used H_2_O_2_ to establish a senescent model for further and easier research. However, there are many differences between induced senescence and natural senescence. Whether our results can be applied to naturally senescent cells in vitro, and even in vivo, remains to be discussed. Secondly, compared with knockdown, shRNA attenuated protein expression enormously. Hence, our results were not perfect. In addition, NAD^+^ is widespread in cells and is associated with various functions, and senescence is a slow and complex process involving many regulators and signaling pathways. Therefore, other signaling pathways may play roles in preventing senescence after the elevation of intracellular NAD^+^ levels. Finally, we found that excessive PJ34 was harmful to EPCs, but the most appropriate concentration remains to be determined.

## Conclusions

PJ34 can improve the function of aging EPCs through PARP1 inhibition, preservation of intracellular NAD^+^ levels, and increased SIRT1 activity.

## Additional file

Additional file 1: **Figure S1.** Identification of EPCs from human umbilical cord blood. Cells were characterized by flow cytometry detection of CD34, VEGFR2, and CD133. (TIF 138 kb)

## Abbreviations

Ad-GFP: Adenoviral vectors containing green fluorescent protein
ADP: Adenosine diphosphate
Ad-sh-SIRT1: SIRT1 short-hairpin RNA
AS: Atherosclerosis
CCK: Cell counting kit
DCFH-DA: Dichloro-dihydro-fluorescein diacetate
EGM: Endothelial cell growth medium
EPC: Endothelial progenitor cell
NAD^+^: Nicotinamide adenine dinucleotide
P: Passage
PAR: Poly ADP-ribose
PARP1: Poly (ADP-ribose) polymerase 1
ROS: Reactive oxygen species
SA-β-gal: Senescence-associated beta-galactosidase
SIRT1: Sirtuin 1
TCA: Tricarboxylic acid
VEGFR2: Vascular endothelial growth factor receptor 2
vWF: Von Willebrand factor
