# Correction to: PARP1 inhibitor (PJ34) improves the function of aging-induced endothelial progenitor cells by preserving intracellular NAD^+^ levels and increasing SIRT1 activity

**DOI:** 10.1186/s13287-018-1019-6

**Published:** 2018-10-25

**Authors:** Siyuan Zha, Zhen Li, Qing Cao, Fei Wang, Fang Liu

**Affiliations:** Department of Geriatrics, Xinhua Hospital, School of Medicine, Shanghai Jiao Tong University, Shanghai, China

## Correction

The original article [1] contains an error regarding the erroneous inclusion of 3 μl as a parameter in the x-axis of Fig. 2c; the correct version of Fig. 2c can instead be seen below.

**Fig. 2:**
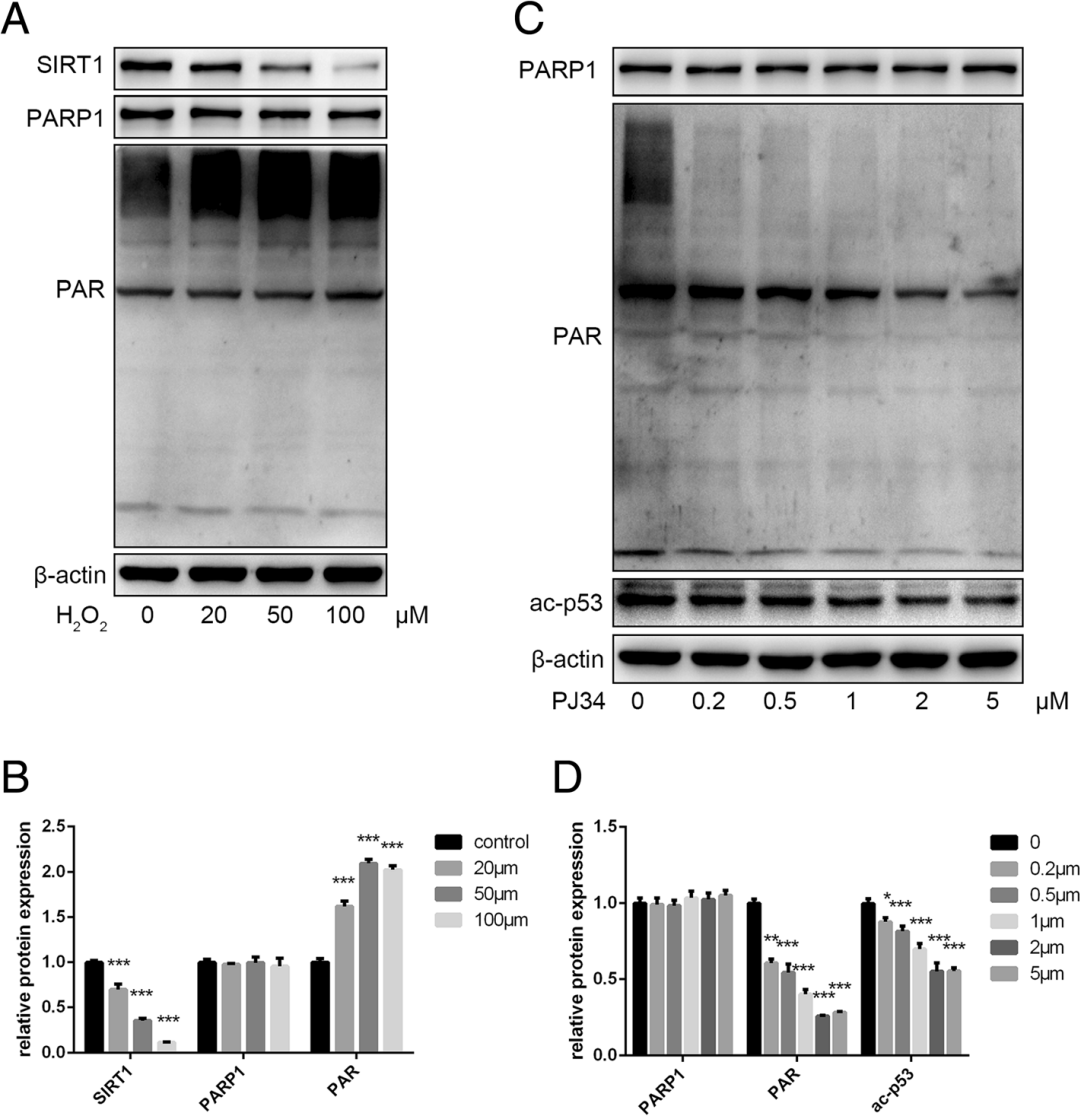
Effects of H_2_O_2_ and PJ34 treatment on EPC protein expression. **a**, **b** Expression of sirtuin 1 (SIRT1), poly (ADP-ribose) polymerase 1 (PARP1), and poly ADP-ribose (PAR) as analyzed by Western blot. **c**, **d** Expression of PARP1, PAR, and acetylated (ac)-p53 as analyzed by Western blot. *P < 0.05, **P < 0.01, ***P < 0.001, versus the control
