# Supplementary figures and images for: PARP1 inhibitor (PJ34) improves the function of aging-induced endothelial progenitor cells by preserving intracellular NAD+ levels and increasing SIRT1 activity
Source: Stem Cell Res Ther. 2018 Aug 23;9:224. doi: 10.1186/s13287-018-0961-7 (PMC6107962; doi:10.1186/s13287-018-0961-7)

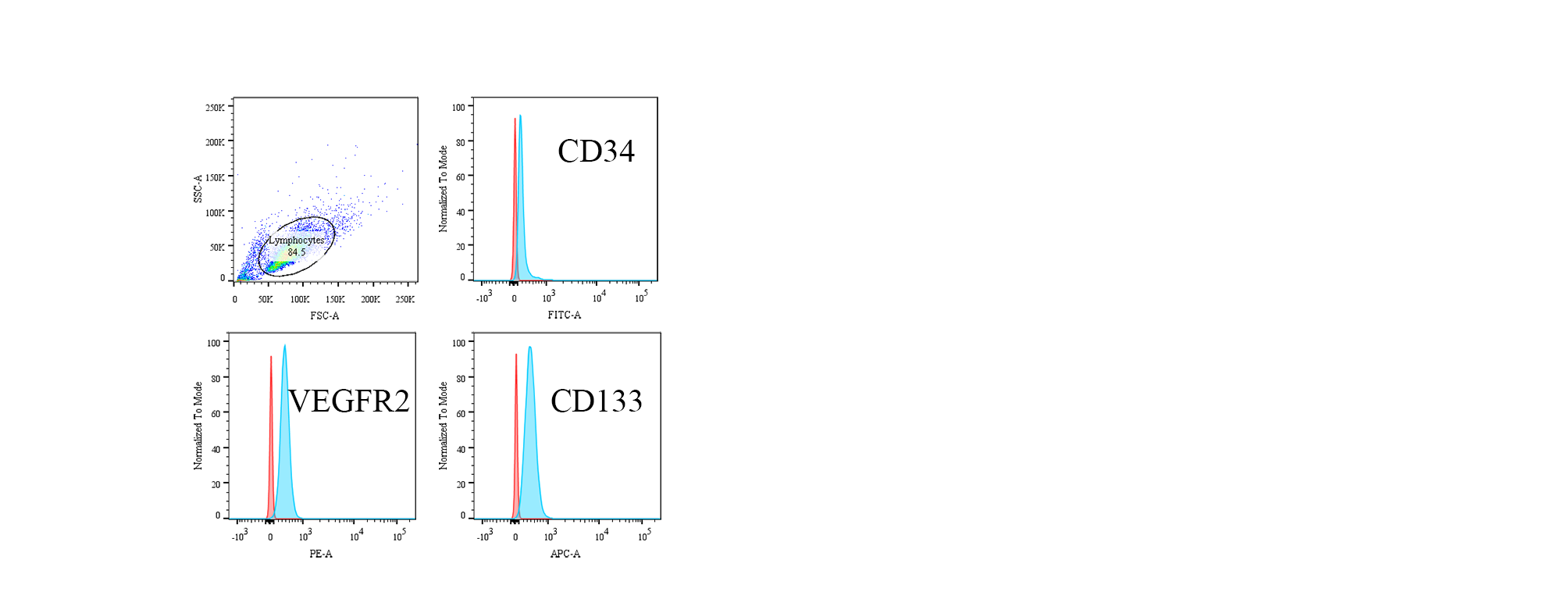

Supplement: Supplementary file 1 — Figure S1. Identification of EPCs from human umbilical cord blood. Cells were characterized by flow cytometry detection of CD34, VEGFR2, and CD133. (TIF 138 kb) [file 13287_2018_961_MOESM1_ESM.tif]
